# Polyunsaturated Fatty Acids and Microbiota Relationship: Implications in Cancer Onset and Treatment

**DOI:** 10.3390/jcm9113490

**Published:** 2020-10-29

**Authors:** Lara Costantini, Nicolò Merendino

**Affiliations:** Department of Ecological and Biological Sciences (DEB), Tuscia University, Largo dell’Università snc, 01100 Viterbo, Italy; merendin@unitus.it

**Keywords:** Omega-3 fatty acids, omega-6 fatty acids, microbiota, microbiome, cancer, tumor, docosahexaenoic acid, DHA, eicosapentaenoic acid, EPA

In these recent years, a growing interest with regard to polyunsaturated fatty acids (PUFAs) and microbiota relationship has been noted. The interest regarding this relationship is mostly fueled by the fact that PUFAs seem to be differently involved in the onset of non-communicable diseases. Indeed, although such pathologies are multi-factorial, their prevalence coincides with the unbalanced ratio between n-3/n-6 PUFAs. In particular, the high intake of dietary n-6 PUFAs, which leads to upper n-6-/n-3 ratios of 4:1, appears to be responsible of the chronic low-grade inflammation, a critical etiological factor for chronic illnesses. However, this topic remains controversial due to discordant results. For this reason, in recent years, various paths have been taken to explain the PUFAs and non-communicable diseases relationship. One of these concerns the microbiota involvement in different non-communicable diseases, and the microbiota and PUFAs mutual relationship was hypothesized and partially demonstrated. Most of the current studies concern dietary PUFAs supplementations in animal models and often without taking into account the final n-6/n-3 PUFA ratio. Overall, these studies have highlighted a positive action of n-3 PUFAs in restoring the eubiosis (i.e., homeostasis of the microbiota populations), especially of long-chain n-3 PUFAs [1] and in particular, when the pathology in question is caused by chronic inflammation. Conversely, when the inflammation is necessary to overcome the pathology, the anti-inflammatory action of n-3 PUFAs could be detrimental [2]. However, for n-6 PUFAs, the first investigations gave mixed results. Only recently, has the elegant work of Kaliannan and co-workers [3] begun to shed light on that issue bypassing the confounding factors of diet. Indeed, it was shown that alteration in the tissue n-6/n-3 PUFA ratio, obtained with transgenic mice able to overproduce n-6 PUFAs or to convert n-6 to n-3 PUFAs, correlates with changes in the gut microbiota populations, and with fecal and serum metabolites. In particular, *Enterobacteriacea* and *Verromicrobiaceae* were the most abundant families in the over-productive n-6 PUFAs genotype, while the *Bifidobacteriacea*, *Desulfovibrionaceae*, and *Bacteroidaceae* families were the most abundant in the over-productive n-3 PUFAs genotype. Furthermore, concurrently, a number of metabolite markers of gut dysbiosis, inflammation, and chronic diseases were elevated in the over-productive n-6 PUFAs genotype and depleted in the over-productive n-3 PUFAs genotype. Among them, elevated levels of 1-methylnicotinamide, as a marker of dysbiosis; cysteine and histidine, as markers of increased gut permeability; and lactate and spermidine, as markers of gut inflammation, have been found [3]. Moreover, concomitant studies showed the mutual relationship that microbiota could have in the PUFAs’ metabolism. Through in vivo stable isotope labeling experiments and a dietary intervention strategy, Kindt and colleagues [4] showed that the acetate molecule (2:0), a short chain fatty acid generated from gut microbial degradation of dietary fiber, is a precursor of long-chain fatty acids synthetized in the liver. In addition, they found that the presence of a gut microbiota increased the desaturation of the palmitate molecule (16:0) by stearoyl-CoA desaturase 1, and elongation of γ-linoleic acid (18:3 n-6) to dihomo-γ-linoleic acid (20:3 n-6) by long-chain fatty acid elongase 5 [4]. Subsequently, in the paper of Miyamoto and co-workers [5], it was proven that in mice fed a high-fat diet, the *Lactobacillus*-colonized gut microbiota converted the n-6 PUFA linoleic acid in the metabolite 10-hydroxy-cis-12-octadecenoic acid, reducing linoleic acid conversion in the inflammatory eicosanoids of the arachidonic acid cascade [5].

Therefore, these first studies laid the foundations to hypothesize an active and reciprocal role of PUFAs and microbiota in relation to chronic diseases. In particular, what role does this relationship play in carcinogenesis? In a first study on a mouse model of azoxymethane-dextran sulfate sodium (AOM-DSS)-induced colorectal cancer, the supplementation of the long-chain n-3 PUFA, eicosapentaenoic acid (EPA), strongly decreased the tumor multiplicity, incidence, and size. Moreover, these effects were concomitant to *Lactobacillus* species enrichment in the gut microbiota populations, counteracting the dysbiosis induced by DSS and facilitating the recovery of a health-promoting layout of the gut microbiota [6]. This was supported by a following in vitro study that showed the role of the long-chain n-3 PUFA docosahexaenoic acid (DHA) in improving the adhesion of *Lactobacilli* to human colonic epithelial cells [7].

Subsequent significant human studies analyzed the relationship of microbiota and n-3 PUFAs in some steps of the carcinogenesis. In the paper of Prossomariti and colleagues [8], in patients with long-standing ulcerative colitis (risky patients of colorectal cancer), EPA supplementation 2 g/daily for 90 days led to improvement in endoscopic and histological inflammation, concurrently with the modulation of the gut microbiota. In particular, the *Parabacteroides* genus, known as diminished in patients with ulcerative colitis, was significantly increased after EPA supplementation. Conversely, *Clostridium* spp. and *Bacteroides*, both known to trigger mucolytic metabolism, were found decreased after EPA supplementation, further contributing to the protection of the epithelium [8]. To support the action of PUFAs in carcinogenesis, in the recent paper of Kim and colleagues [9], a metabolomic profile of stool samples was performed in healthy people, in patients with colorectal adenomas (i.e., precancerous lesions of colorectal cancer), and in colorectal cancer patients. The authors found that both n-3 and n-6 PUFA metabolites were elevated in adenoma patients compared to the control, and this perturbation was also found in the carcinoma group, suggesting that imbalances in PUFAs seem to play a significant role in the carcinogenesis process. Moreover, these perturbations were significantly correlated with multiple bacteria genera (*Clostridium*, *Dehalobacterium*, *Ruminococcus*, *Oscillospira*, *Bacteroides*). However, even if this study recorded the relationship between carcinogenesis, microbiota, and PUFAs, it has several limitations such as the possibility of not discriminating between n-3 and n-6 PUFA metabolites, and the unknown absolute concentrations [9]. Finally, in the paper of Horigome and co-workers [10], positive associations between n-3 PUFA levels (EPA and DHA) in blood and some gut bacterial taxa (*Actinobacteria*, *Bacteroidetes*, and *Bifidobacterium*) were found in breast cancer survivors, but only without a history of chemotherapy [10].

In conclusion, recent studies have begun to explain the correlation between the PUFAs–microbiota–cancer triad, but the topic is completely open, especially in relation to n-6 PUFAs and tumors outside the intestinal district, where studies are almost absent. The multidisciplinary approach combining metagenomics with metabolomics as well as metatrascriptomics and metaproteomics may be the solution to understanding not only the microbiota composition following PUFA intervention, but also its activity.
